# (S)-(-)-blebbistatin O-benzoate has the potential to improve atopic dermatitis symptoms in NC/Nga mice by upregulating epidermal barrier function and inhibiting type 2 alarmin cytokine induction

**DOI:** 10.1371/journal.pone.0302781

**Published:** 2024-05-07

**Authors:** Shunya Sahara, Ayumi Ueno, Natsuki Wakita, Miki Iwai, Junki Uda, Koich Nakaoji, Kazuhiko Hamada, Akito Maeda, Yasufumi Kaneda, Manabu Fujimoto

**Affiliations:** 1 Research and Development Division, PIAS Corporation, Kobe, Hyogo, Japan; 2 Office of Management and Planning, Osaka University, Suita, Osaka, Japan; 3 Vice President Office, Osaka University, Suita, Osaka, Japan; 4 Department of Dermatology, Graduate School of Medicine, Osaka University, Suita, Osaka, Japan; Kyungpook National University School of Medicine, REPUBLIC OF KOREA

## Abstract

Atopic dermatitis is a multi-pathogenic disease characterized by chronic skin inflammation and barrier dysfunction. Therefore, improving the skin’s ability to form an epidermal barrier and suppressing the production of cytokines that induce type 2 inflammatory responses are important for controlling atopic dermatitis symptoms. (-)-Blebbistatin, a non-muscle myosin II inhibitor, has been suggested to improve pulmonary endothelial barrier function and control inflammation by suppressing immune cell migration; however, its efficacy in atopic dermatitis is unknown. In this study, we investigated whether (S)-(-)-blebbistatin O-benzoate, a derivative of (-)-blebbistatin, improves dermatitis symptoms in a mite antigen-induced atopic dermatitis model using NC/Nga mice. The efficacy of the compound was confirmed using dermatitis scores, ear thickness measurements, serum IgE levels, histological analysis of lesions, and filaggrin expression analysis, which is important for barrier function. (S)-(-)-Blebbistatin O-benzoate treatment significantly reduced the dermatitis score and serum IgE levels compared to those in the vehicle group (*p* < 0.05). Furthermore, the histological analysis revealed enhanced filaggrin production and a decreased number of mast cells (*p* < 0.05), indicating that (S)-(-)-blebbistatin O-benzoate improved atopic dermatitis symptoms in a pathological model. *In vitro* analysis using cultured keratinocytes revealed increased expression of filaggrin, loricrin, involucrin, and ceramide production pathway-related genes, suggesting that (S)-(-)-blebbistatin O-benzoate promotes epidermal barrier formation. Furthermore, the effect of (S)-(-)-blebbistatin O-benzoate on type 2 alarmin cytokines, which are secreted from epidermal cells upon scratching or allergen stimulation and are involved in the pathogenesis of atopic dermatitis, was evaluated using antigens derived from mite feces. The results showed that (S)-(-)-blebbistatin O-benzoate inhibited the upregulation of these cytokines. Based on the above, (S)-(-)-blebbistatin O-benzoate has the potential to be developed as an atopic dermatitis treatment option that controls dermatitis symptoms by suppressing inflammation and improving barrier function by acting on multiple aspects of the pathogenesis of atopic dermatitis.

## Introduction

Atopic dermatitis (AD) is a multi-etiological disease. Many patients have a family or medical history of allergic diseases or a predisposition to atopy, such as a tendency to produce IgE antibodies, which results in chronic skin inflammation and abnormal barrier function [1]. Its prevalence is increasing annually, and approximately 230 million people are estimated to be affected worldwide [2]. The symptoms of dermatitis often continue into adulthood, and the stress of exacerbating symptoms and anxiety about interpersonal relationships adversely affect daily life (professional work and schoolwork) and considerably reduce the quality of life of the affected patients [2]. Topical treatment with anti-inflammatory drugs, mainly steroids, and moisturizers, such as petroleum jelly, is the mainstay of dermatitis treatment [1, 3]. In severe cases, the biologic drugs dupilumab and oral Janus kinase inhibitors, which are molecularly targeted drugs, are now available; however, their use is limited owing to high drug costs and the need to take measures against side effects [4, 5]. AD is a difficult-to-control skin disease characterized by chronic relapsing inflammation that requires long-term treatment [3, 6]. Therefore, superior treatment options with minimal adverse effects need to be developed. In recent years, topical agents such as delgocitinib and difamilast have been developed to increase the options for topical agents [7, 8].

Controlling inflammation and improving barrier function are important for the treatment of AD [1]. The material-permeable barrier of the stratum corneum of the epidermis is formed by lamellar structures containing ceramides, fatty acids, and tight junctions [9]. In addition, a natural moisturizing factor, a degradation product of filaggrin, contributes to moisture retention as a moisturizing component of the stratum corneum [10]. Decreased ceramide content and filaggrin expression have been reported in AD lesions, indicating that these factors contribute to abnormal barrier function [11, 12]. Impaired barrier function facilitates the entry of antigens into the interior of the skin, and injury of keratinocytes by these antigens induces the production of alarmin cytokines such as thymic stromal lymphopoietin (TSLP) and interleukin (IL)-33 [13]. These cytokines activate group 2 innate lymphoid cell (ILC2) and Th2 cells, inducing a type 2 immune response in the skin [14, 15], and IL-4 and IL-13 produced by ILC2 and Th2 cells inhibit the expression of epidermal differentiation-related genes, including filaggrin, in keratinocytes, causing further skin barrier degradation [16–18]. Therefore, it is important to restore the ability of keratinocytes to form the skin barrier and inhibit the production of cytokines that induce type 2 immune responses to control the skin inflammatory symptoms of AD. (-)-Blebbistatin is a non-muscle myosin II inhibitor that affects the function of the cytoskeletal system in cells [19]. (-)-Blebbistatin and its derivatives have been reported to have potential applications in the treatment of various conditions including wound healing and fibrosis [20]. It has also been suggested that in acute lung injury (-)-blebbistatin improves endothelial barrier dysfunction of the lung [21]. However, the ameliorative effects of (-)-blebbistatin and its derivatives on AD are unknown.

In this study, we report a detailed *in vivo* and *in vitro* investigation of the effects and mechanisms of action of (-)-blebbistatin and its derivatives on atopic symptoms.

## Materials and methods

### Ethics statement

All animal experiments were performed in strict accordance with the Guidelines for Animal Experiments at the Osaka University Graduate School of Medicine. The experimental protocols were approved by the Animal Experiments Committee of Osaka University (Permit number: 02-080-000). All procedures were performed under isoflurane inhalation anesthesia, and every effort was made to minimize the suffering of the mice.

### Animals

Thirty-two female NC/Nga mice (8 weeks old) were obtained from Japan SLC (Hamamatsu, Japan). Female mice were used to avoid the risk of damage to the observation site due to fights between mice in the breeding cage [22, 23]. The mice were housed in a sterile animal facility with the temperature controlled at 23°C ± 1.5°C and relative humidity at 50% under a 12-:12-h light/dark cycle and free access to standard laboratory diet and water. During the experiment, the health of the mice was observed once a day to check for the presence of the auricular reflex, auditory reflex, and pain reflex. Mice that did not respond to any of these measures were to be immediately terminated from the study and euthanized. However, no mice met the above humane endpoint criteria during the experiment. On the final day of the experiment (Day 35), all mice were euthanized by exsanguination, and whole blood was collected from the posterior vena cava under isoflurane anesthesia.

### House dust mite-induced AD model

Thirty-two NC/Nga mice were injected intradermally with 20 μg of Mite Extract Dp (Cosmo Bio, Tokyo, Japan) dissolved in saline on the ventral side of their right ears on Days 0, 3, 7, 10, and 14, to induce dermatitis [22]. On Day 15, the thickness and skin symptom score of the right ear were measured and the mice were divided into four groups using the stratified allocation method to ensure an equal mean value for each group (N = 8 in each group). (-)-Blebbistatin (TargetMol Chemicals, Boston, MA), (S)- (-)-blebbistatin O-benzoate (Toronto Research Chemicals, Toronto, Canada) (5 mg/mL in acetone/methanol), or vehicle was administered to the lesion site for 20 days, and changes in the dermatitis score and ear thickness were evaluated. Moreover, 0.1% FK506 (Selleck Chemicals, Houston, TX, USA) was used as the positive control.

### Dermatitis score and ear thickness measurement

The severity of right auricular dermatitis was assessed macroscopically. Severity was assessed using four scales: redness/erythema, hemorrhage/clots, crust/excoriation, and edema/induration. Each scale was graded from 0 to 3 (0, absent; 1, mild; 2, moderate; and 3, severe), and the total values were calculated (Table 1) [23]. The thickness of the right auricle was measured using an upright gauge (R1-A; Ozaki MFG Co., Ltd., Tokyo, Japan) under isoflurane anesthesia. Based on the measured value at the time of grouping, the change in auricle thickness was calculated.

**Table 1:** Dermatitis score of auricular [23].

Criteria	Absent = 0	Mild = 1	Moderate = 2	Severe = 3
**Redness/erythema**	None	Redness along blood vessels, mainly dilation of blood Vessels	Redness spreading from blood vessels	Redness covering more than half of the auricular area
**Hemorrhage/clots**	None	One or two spots of hemorrhage/clots	Three or more spots of hemorrhage/clots or one or more hemorrhage/clot areas	Hemorrhage/clots covering more than half of the auricular area
**Crust/excoriation**	None	A few spots of crust/excoriation or one crust/excoriation area	Multiple spots of crust/excoriation or two or more crust/excoriation areas	Crust/excoriation covering more than 1/3 of the auricular area
**Edema/induration** ^**#**^	None	Can be bent with feeling the core, more than one point of edema/induration	Some hardened portions cannot be bent, extensive edema/induration	Edema/induration covering more than half of the auricular area

^#^Severity of edema/induration was assessed by slowly bending the auricle with the fingers.

### Serum IgE measurement

Under isoflurane anesthesia, the mice were euthanized by collecting whole blood from the posterior vena cava. The collected blood samples were allowed to stand at room temperature (15°C to 25°C) for 30 min, and serum was separated by centrifugation (1,800 × *g*, 10 min, 4°C). The serum was stored at -80°C until analysis. The serum IgE concentration was quantified using a mouse IgE EIA kit (Yamasa, Choshi, Japan) according to the manufacturer’s instructions.

### Histological analysis

The right auricles of euthanized mice were harvested and fixed in 10% neutral-buffered formalin. Tissues were then embedded in paraffin and sectioned (4-μm thick). Skin sections were stained with hematoxylin and eosin (H&E) or toluidine blue to observe epidermal inflammation and mast cell infiltration. Epidermal thickness was quantified by measuring five locations on H&E-stained images of each tissue using the ImageJ software (National Institutes of Health, NIH, Bethesda, MD, USA, http://rsb.info.nih.gov/ij/). Dermal mast cells were quantified by randomly selecting six areas in high-power fields for each tissue and counting the number of toluidine blue-positive cells. To observe filaggrin expression in the epidermis, deparaffinized sections were incubated with Filaggrin Polyclonal Antibody (1:1000 Clone: Poly19058; BioLegend, San Diego, CA, USA) overnight at 4°C. Secondary antibody (peroxidase-labeled anti-rabbit IgG polyclonal antibody) and 3,3’-diaminobenzidine-4HCl staining were performed according to the manufacturer’s protocol (#424144; Nichirei, Tokyo, Japan).

### Cell culture

Normal human epidermal keratinocytes (NHEKs; KK-4009) were purchased from KURABO Industries Ltd. (Osaka, Japan) and cultured in a medium for normal human epidermal keratinocyte proliferation (HuMedia-KG2; KURABO Industries Ltd.). The cells were passaged at 70%–80% confluence, and each experiment was conducted using cells within three passages.

### Cell viability assay

The viability of keratinocytes was measured using the 3-(4,5- dimethylthiazol-2-yl)-5-(3-carboxymethoxyphenyl)-2-(4-sulfophenyl)-2H-tetrazolium (MTS) assay as described previously [24]. In brief, keratinocytes were seeded at a density of 5.0 × 10^3^ cells/well in a 96-well plate and maintained at 37°C for 48 h. The cells were then treated with 1, 2, 5, 10, or 20 μM (S)-(-)-blebbistatin O-benzoate for 48 h. The medium in each well was removed and 100 μL of CellTiter 96 One Solution reagent (Promega, Madison, WI, USA) diluted 6-fold in culture medium was added; the cells were incubated at 37°C for 2 h and absorbance of the samples was measured at 490 nm. As a control, the proliferation activity of cells incubated with a normal medium was measured. The relative number of cells was calculated by normalizing the data to the control value. The experiments were conducted three times independently, and each repetition was performed with three samples from each group.

### Keratinocyte differentiation assay

Keratinocytes were seeded at a density of 6.0 × 10^4^ cells/well in a 12-well plate and maintained at 37°C for 48 h. The cells were then treated with 5 or 10 μM (S)-(-)-blebbistatin O-benzoate for 48 h, and cell morphology was observed using an optical microscope (IX 70; Olympus, Tokyo, Japan). The morphology of keratinocytes was compared with that of cells incubated for 48 h in a normal medium and 1.35 mM calcium-containing medium.

### Expression of skin barrier-functional molecules in keratinocytes

Keratinocytes were seeded at a density of 6.0 × 10^4^ cells/well in a 12-well plate and maintained at 37°C for 72 h. The cells were treated with 5, 10, or 20 μM (S)-(-)-blebbistatin O-benzoate for 72 h and gene expression was assessed. As a control, gene expression in cells incubated with HuMedia-KG2 only was measured. The experiments were conducted three times independently, and each repetition was performed with three samples from each group.

### Suppression of cytokine production by mite antigen stimulation in keratinocytes

Keratinocytes were seeded at a density of 6.0 × 10^4^ cells/well in a 12-well plate and maintained at 37°C for 72 h. The cells were stimulated with 12.5 μg/mL Mite-Dp Feces Crude Extract (Cosmo Bio), 20 ng/mL TNF-α (R&D Systems, Minneapolis, MN, USA), and 100 ng/mL IL-4 (R&D Systems) in the presence of 5 or 10 μM (S)-(-)-blebbistatin O-benzoate for 24 h and gene expression was assessed [25–27]. Cells stimulated with mite feces antigen were used as a control to compare gene expression levels. The experiments were conducted three times independently, and each repetition was performed with three samples from each group.

### Gene expression (reverse transcription-polymerase chain reaction [RT-PCR])

Real-time RT-PCR was performed for the quantitative detection of gene expression in keratinocytes. Total RNA was extracted using the PureLink RNA Mini kit (Thermo Fisher Scientific, Waltham, MA, USA) and reverse-transcribed using ReverTra Ace qPCR RT Master Mix (TOYOBO, Osaka, Japan) according to the manufacturer’s instructions. Reactions were performed using Power SYBR Green PCR Master Mix (Applied Biosystems, Foster City, CA, USA) and a 7300 real-time PCR system (Applied Biosystems). Primer sequences used in these experiments are listed in Table 2. Expression of each gene was normalized to that of glyceraldehyde-3-phosphate dehydrogenase.

**Table 2:** List of primers used in this study.

Used Gene		Primer sequence (5′ → 3′)	Reference
***FLG***	F	CAGACAATCAGGCACTCGTCA	[28]
	R	ACTGGACCCTCGGTTTCCAC	
***IVL***	F	TCCTCCAGTCAATACCCATCAG	[29]
	R	CAGCAGTCATGTGCTTTTCCT	
***LOR***	F	GGCTGCATCTAGTTCTGCTGTTTA	[30]
	R	CAAATTTATTGACTGAGGCACTGG	
***SPTLC2***	F	CCTGCTCTTGTTGGCAAAGG	[31]
	R	GCTCCCAGAACCAGTGATGC	
***SMPD1***	F	TGGCTCTATGAAGCGATGGC	[32]
	R	TTGAGAGAGATGAGGCGGAGAC	
***GBA***	F	GCTAGGCTCCTGGGATCGAG	[33]
	R	GTTCAGGGCAAGGTTCCAGTC	
***TSLP***	F	GCCATGAAAACTAAGGCTGC	[34]
	R	CGCCACAATCCTTGTAATTG	
***IL-33***	F	GTGACGGTGTTGATGGTAAGAT	[35]
	R	AGCTCCACAGAGTGTTCCTTG	
***IL-1α***	F	AACCAGTGCTGCTGAAGGA	[36]
	R	TTCTTAGTGCCGTGAGTTTCC	
***IL-1β***	F	ACAGATGAAGTGCTCCTTCCA	[37]
	R	GTCGGAGATTCGTAGCTGGAT	
***GAPDH***	F	GCTCTCTGCTCCTCCTGTTC	[29]
	R	ACGACCAAATCCGTTGACTC	

F, forward; R, reverse.

### Statistical analyses

Results are expressed as the mean ± standard error (SE). For the analysis of *in vivo* studies, statistically significant differences between the control and other groups were determined using Steel’s test or the Brunner–Munzel test. Analysis of *in vitro* studies used Dunnett’s test after confirming normality and homoscedasticity; * and ** correspond to *p* < 0.05 and *p* < 0.01, respectively. These analyses were performed using BellCurve for Excel v3.21 (SSRI, Tokyo, Japan).

## Results

### Topical administration of (S)-(-)-blebbistatin O-benzoate reduces dermatitis symptoms in NC/Nga mice

**Fig 1:**
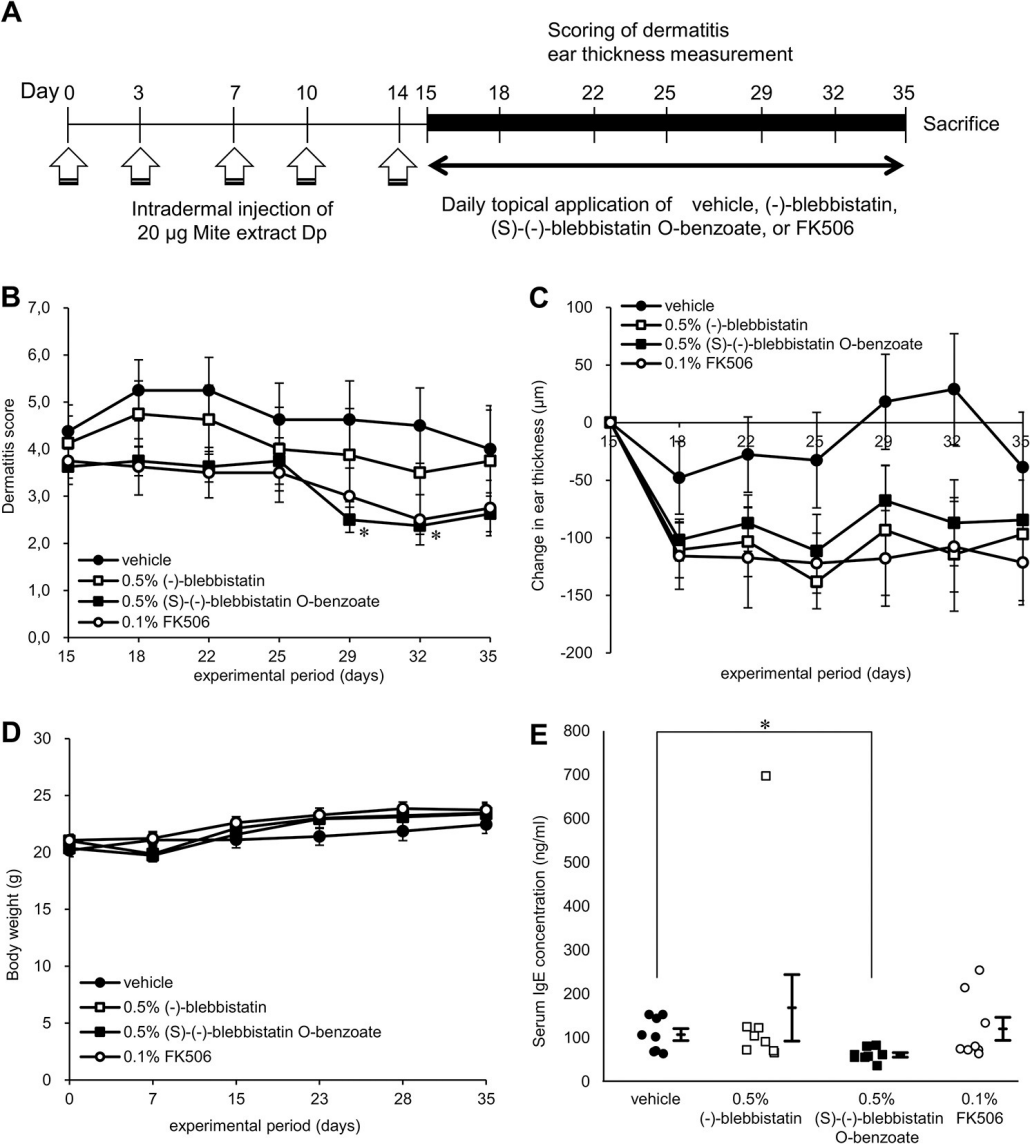
Treatment with (S)-(-)-blebbistatin O-benzoate improves mite antigen-induced AD-like dermatitis in NC/Nga mice. (A) The experimental scheme for induction of house dust mite-induced AD-like skin inflammation and treatment (n = 8 in each group). (B) Dermatitis score and (C) Ear thickness were measured on Days 15, 18, 22, 25, 29, 32, and 35 of the experimental period in the right ear. (S)-(-)-blebbistatin O-benzoate showed a significant reduction in dermatitis score compared to the vehicle. (D) Weight changes of mice were measured once a week during the study period. (E) On Day 35, serum was collected and IgE levels were measured by enzyme-linked immunosorbent assay (ELISA). (S)-(-)-blebbistatin O-benzoate showed a significant decrease compared to the vehicle. Data represent mean ± SE of all experiments, *p < 0.05 vs. vehicle.

The therapeutic effects of (-)-blebbistatin and its analog (S)-(-)-blebbistatin O-benzoate on auricular skin lesions were investigated in an AD model using NC/Nga mice (Fig 1A). The results showed that the dermatitis scores of (S)-(-)-blebbistatin O-benzoate-treated mice were significantly reduced compared to those of the vehicle on Days 29 (vehicle: 4.6 ± 0.8, (S)-(-)-blebbistatin O-benzoate: 2.5 ± 0.3, *p* = 0.0462) and 32 (vehicle: 4.5 ± 0.8, (S)-(-)-blebbistatin O-benzoate: 2.4 ± 0.2, *p* = 0.0393) (Fig 1B). On the contrary, (-)-blebbistatin showed a similar trend but no significant difference. The change in ear thickness after treatment was not significantly different in either group compared to the vehicle but tended to decrease to the same level as that of the positive control FK506 (Fig 1C). During the study period, changes in the body weight of the mice were not affected by drug administration (Fig 1D). On the last day of the study (Day 35), whole blood was collected from the mice and serum IgE levels were measured. As a result, (S)-(-)-blebbistatin O-benzoate treatment significantly reduced serum IgE levels compared to the vehicle (vehicle: 107.3 ± 12.7 ng/mL, (S)-(-)-blebbistatin O-benzoate: 61.2 ± 4.9 ng/mL, *p* = 0.0174) (Fig 1E).

**Fig 2:**
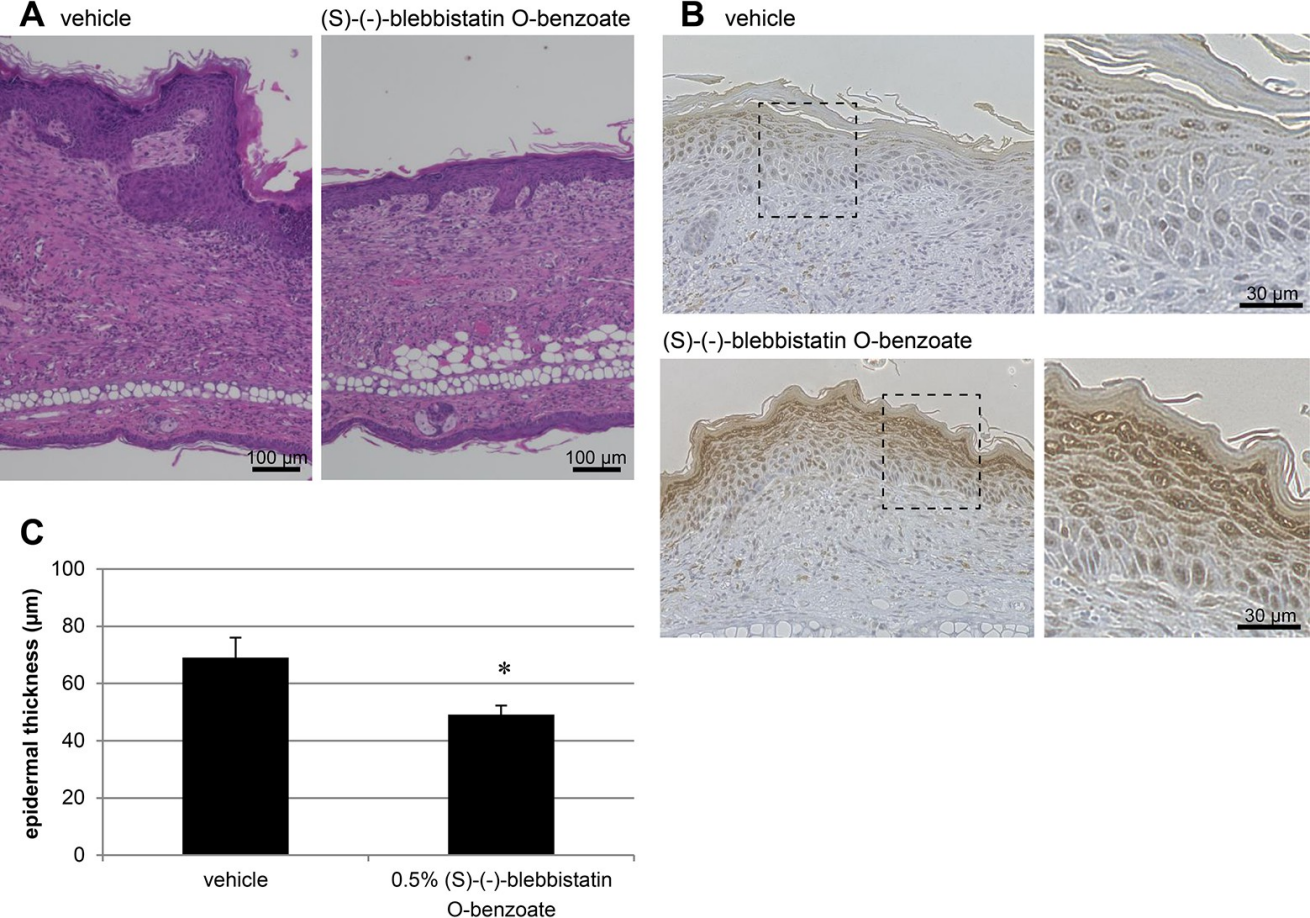
(S)-(-)-blebbistatin O-benzoate improves skin barrier function in AD-like lesions of NC/Nga mice. Histological observation of auricular tissues on Day 35. (A) Sections recovered from vehicle-and (S)-(-)-blebbistatin O-benzoate-treated mice were stained with hematoxylin and eosin. Scale bar = 100 μm. (B) Immunostaining of filaggrin (FLG). Scale bar = 30 μm. FLG expression in the epidermis was enhanced by (S)-(-)-blebbistatin O-benzoate treatment compared to the vehicle treatment. The data are representative of each group. (C) Epidermal thickness of each tissue was measured based on H&E staining images and evaluated quantitatively (n = 8 per group). (S)-(-)-blebbistatin O-benzoate treatment led to a significant reduction in epidermal thickening. Data represent mean ± SE, *p < 0.05 vs. vehicle.

Furthermore, auricular tissues from the vehicle and (S)- (-)-blebbistatin O-benzoate groups were collected and the expression of filaggrin in the epidermis of the lesional skin was observed by antibody staining. Filaggrin staining was enhanced in the skin of mice treated with (S)-(-)-blebbistatin O-benzoate compared to the vehicle (Fig 2B). Significant improvement in epidermal thickening was also observed with (S)-(-)-blebbistatin O-benzoate treatment (vehicle: 69.1 ± 7.0 μm, (S)-(-)-blebbistatin O-benzoate: 49.1 ± 3.2 μm, *p* = 0.0173) (Fig 2C).

**Fig 3:**
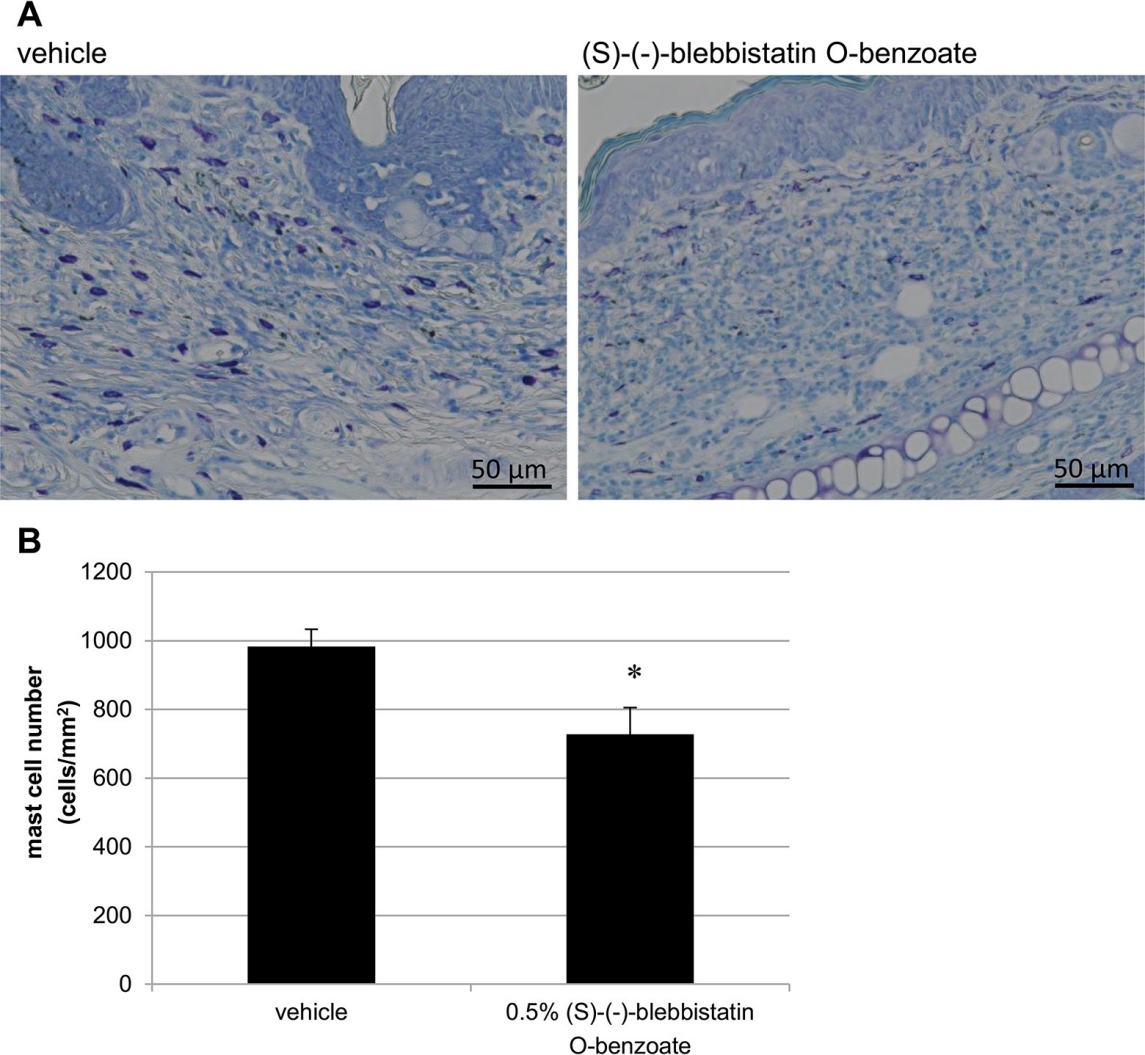
(S)-(-)-blebbistatin O-benzoate reduces mast cell infiltration in AD-like lesions of NC/Nga mice. (A) Toluidine blue staining of auricular tissue of vehicle and (S)-(-)-blebbistatin O-benzoate on Day 35. Scale bar = 50 μm. (B) The number of mast cells stained with toluidine blue was measured and quantitatively evaluated in six areas from each tissue (n = 8 per group). (S)-(-)-blebbistatin O-benzoate treatment significantly decreased mast cell numbers. Data represent mean ± SE, *p < 0.05 vs. vehicle.

In addition, the skin sections were stained with toluidine blue, and the number of mast cells infiltrating the skin tissue was counted. As a result, a significant reduction in the number of mast cells was observed in the (S)- (-)-blebbistatin O-benzoate group compared to the vehicle (vehicle: 982.8 ± 56.7 cells/mm^2^, (S)-(-)-blebbistatin O-benzoate: 727.8 ± 77.4 cells/mm^2^, *p* = 0.0180) (Fig 3).

### Effects of (S)-(-)-blebbistatin O-benzoate on the expression of skin barrier function genes in keratinocytes

**Fig 4:**
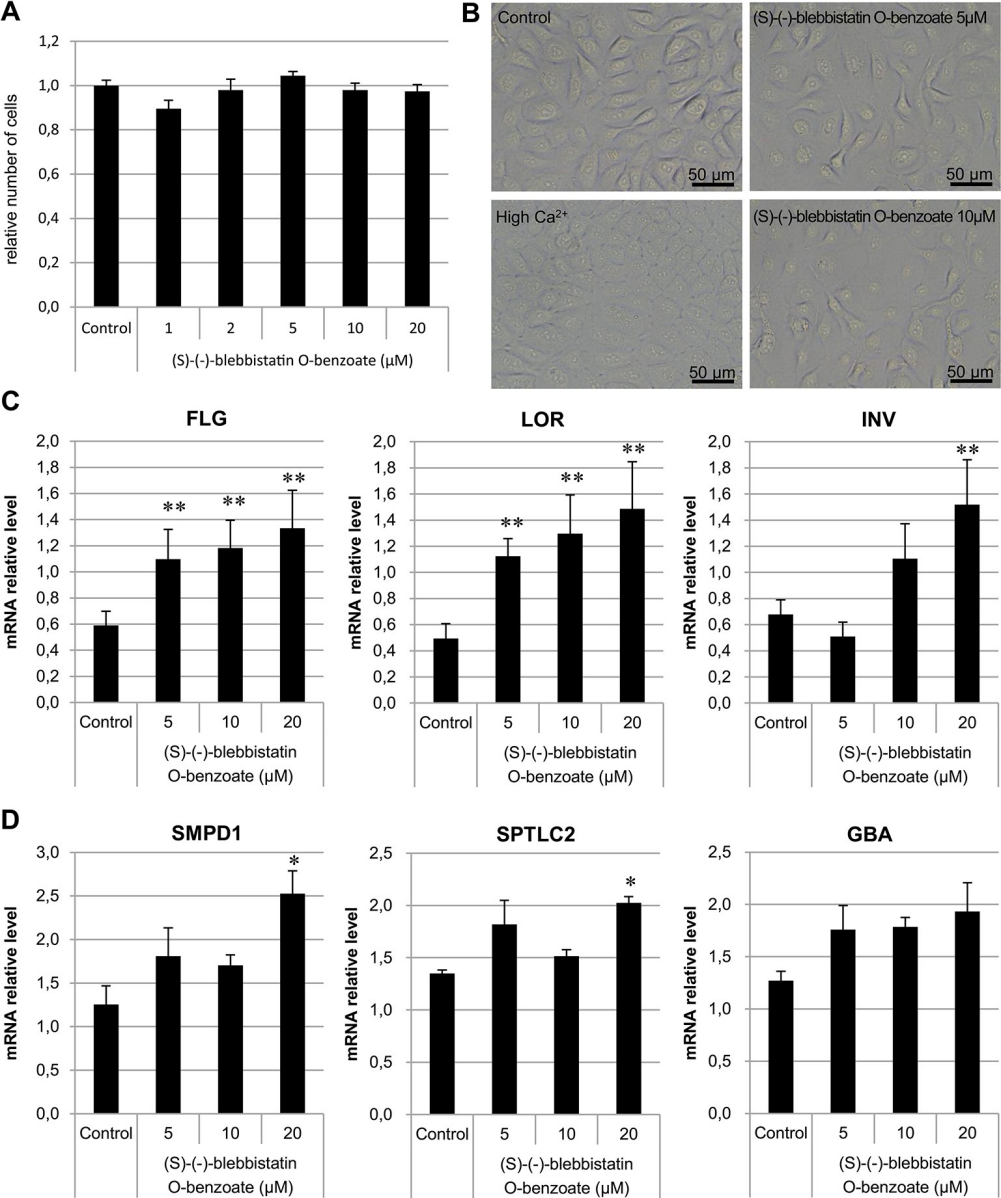
(S)-(-)-blebbistatin O-benzoate upregulates expression of skin barrier function genes in keratinocytes. (A) Viability of keratinocytes exposed to (S)-(-)-blebbistatin O-benzoate for 48 h. (B) Morphology of keratinocytes differentiation incubated Ca^2+^, (S)-(-)-blebbistatin O-benzoate, or normal medium for 48 h. (C) Gene expression of epidermal barrier-related molecules FLG, loricrin (LOR), involucrin (INV), and (D) ceramide production pathway-related genes *SMPD1*, *SPTLC2*, and *GBA* in keratinocytes incubated with (S)-(-)-blebbistatin O-benzoate for 72 h. Data represent mean ± SE (n = 3/group) and are average values of three independent experiments, *p < 0.05 and **p < 0.01 vs. PBS control.

The toxicity of (S)-(-)-blebbistatin O-benzoate on NHEKs was investigated using the MTS assay. The changes in the viability of keratinocytes were negligible after 48 h of exposure to (S)-(-)-blebbistatin O-benzoate (Fig 4A). Observation of cell morphological changes showed that epidermal cells exhibited a moderately differentiated-like morphology compared to the controls and high-calcium treatment (Fig 4B). Real-time PCR was used to determine whether (S)-(-)-blebbistatin O-benzoate affects the expression of skin barrier-related genes in keratinocytes. The results showed that the expression of filaggrin (FLG), loricrin (LOR), and involucrin (INV), which play central roles in epidermal differentiation and barrier function, significantly increased (Fig 4C). In addition, upregulation of several ceramide production pathway-related genes, sphingomyelin phosphodiesterase 1 (*SMPD1*) and serine palmitoyltransferase long-chain base subunit 2 (*SPTLC2*), was observed (Fig 4D) [38].

### (S)-(-)-blebbistatin O-benzoate inhibits mite antigen stimulation-induced cytokine production in keratinocytes

**Fig 5:**
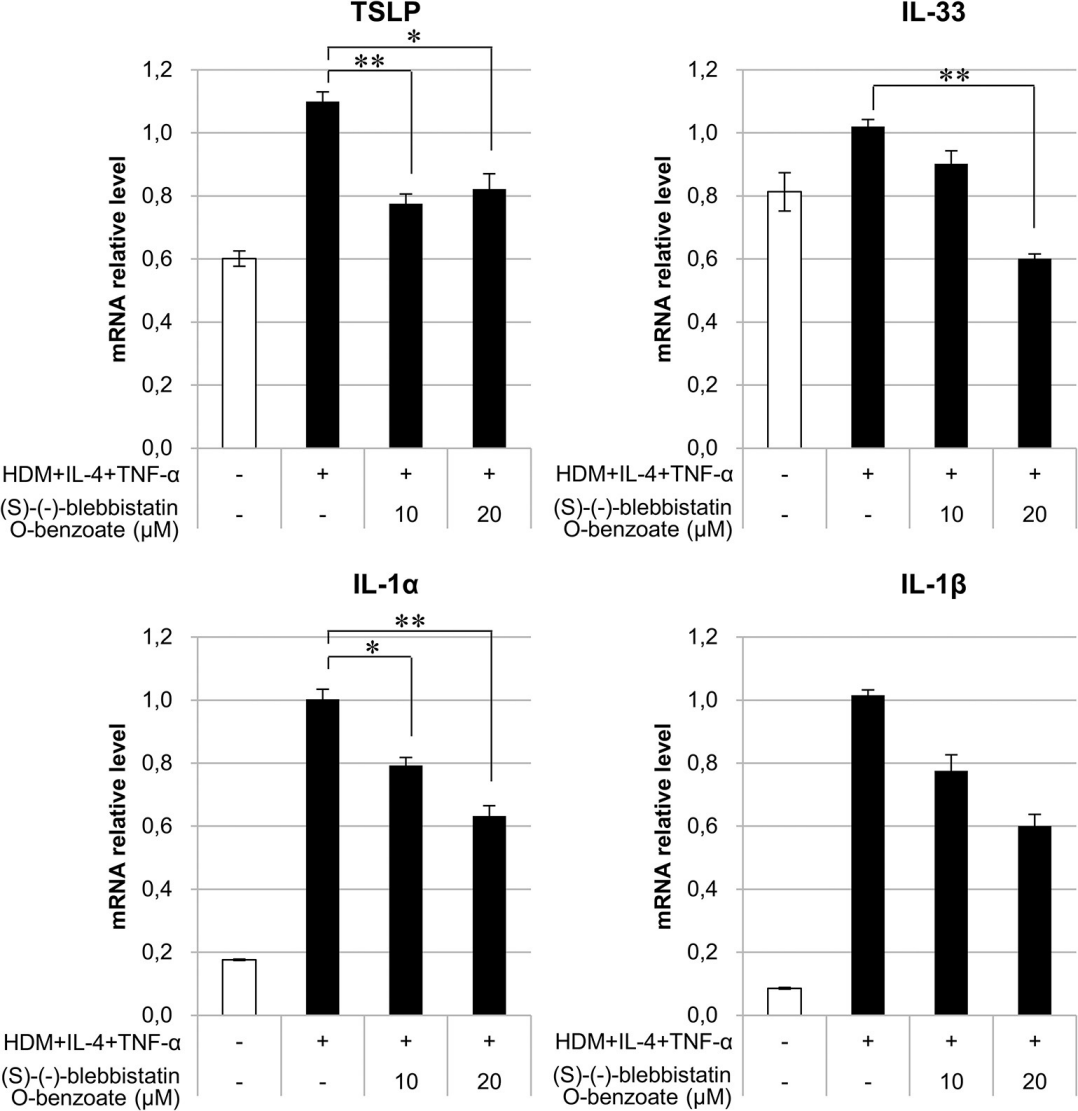
(S)-(-)-blebbistatin O-benzoate suppressed type 2 alarmin cytokine gene in keratinocytes stimulated by mite feces-derived antigen. mRNA expression levels of *TSLP*, *IL-33*, *IL-1α*, and *IL-1β* were measured in keratinocytes with stimulation with mite feces-derived antigen, IL-4, and TNF-α for 24 h. Treatment of cells with (S)-(-)-blebbistatin O-benzoate simultaneously with the above stimulation significantly suppressed gene expression of *TSLP*, *IL-33*, and *IL-1α*. Data represent mean ± SE (n = 3/group) and are average values of three independent experiments, *p < 0.05 and **p < 0.01 vs. HDM stimulation control. HDM, house dust mite feces-derived antigen.

Exposure of keratinocytes to antigens derived from mite excreta is known to increase the expression of cytokines, such as TSLP and IL-33, which induce type 2 immune responses. In this study, we stimulated keratinocytes with a combination of a mite feces-derived antigen, IL-4, and TNF-α to further enhance cytokine expression [27] and investigated the effect of (S)-(-)-blebbistatin O-benzoate on the expression of type 2 alarmin cytokines. As a result, it was revealed that (S)-(-)-blebbistatin O-benzoate suppressed the expression of *TSLP*, *IL-33*, and *IL-1α* which were increased by mite feces-derived antigens (Fig 5).

## Discussion

(-)-Blebbistatin is a non-muscle myosin II inhibitor that affects cytoskeletal function [19]. (-)-Blebbistatin and its derivatives have been reported to have potential applications in the treatment of various diseases, including neurodegenerative disorders, cancer, wounds, and fibrosis [20]. It has also been suggested that the activation of myosin II, a target factor of (-)- blebbistatin, affects the motility of immune cells, such as dendritic cells, and is an important factor in regulating immunity and inflammation [39]. However, the ameliorative effects of (-)-blebbistatin and its derivatives on AD remain unknown.

In the present study, we found that (S)-(-)-blebbistatin O-benzoate, a derivative of (-)-blebbistatin, improved the symptoms of dermatitis in a pathological model of AD created using NC/Nga mice. Skin barrier dysfunction is considered to be a cause of AD [1]. Filaggrins produced by keratinocytes play an important role in the skin barrier function [12]. Filaggrins are stored as profilaggrin in keratohyalin granules in the granular layer and are degraded via keratinization to filaggrin monomers, which promote the formation of a strong stratum corneum barrier by aggregating keratin fibers in the stratum corneum, and upon further degradation, they also function as a natural moisturizing factor [10]. It has been reported that filaggrin production is decreased in AD lesions with or without genetic mutations [16]. In the present study, (S)-(-)-blebbistatin O-benzoate promoted the differentiation of keratinocytes and increased the expression of filaggrin, which is important for barrier function formation, in cultured cells. This effect was also evident from the results of tissue staining of the lesions of AD model mice. (-)-Blebbistatin stimulates the aryl hydrocarbon receptor (AHR) of liver-derived Hepa-1 [40]. Therefore, epidermal differentiation of keratinocytes induced by (S)-(-)-blebbistatin O-benzoate may be due to AHR-mediated signaling. As the activation of the AHR/AHR-nuclear translocator system in the skin promotes skin barrier function and accelerates epidermis differentiation, AHR agonists have been reported to have therapeutic effects on AD [41, 42]. Other studies have also reported that compounds that promote filaggrin expression in keratinocytes reduce the incidence of AD [43, 44]. Other known factors that contribute to skin barrier function include intercellular lipids. Normally, the stratum corneum is filled with intercellular lipids, mainly ceramide, but it has been reported that the ceramide content is abnormally decreased in lesions of patients with AD, resulting in impaired water retention capacity of the stratum corneum [11]. In this study, (S)-(-)-blebbistatin O-benzoate treatment also increased the expression of ceramide production pathway-related genes in keratinocytes, indicating that it may contribute to improving barrier function and stratum corneum water retention. It has been shown that lesions in patients with AD produce cytokines such as TSLP and IL-33 from epidermal cells upon scratching or contact with allergens such as mites, inducing type 2 immune responses [14, 15]. The induction of type 2 immune cells leads to further skin barrier dysfunction and the development of chronic AD symptoms. Cytokines, such as IL-4, produced by Th2 cells, activate B cells and promote IgE production [45]. Secreted IgE activates mast cells in the tissues and causes allergic symptoms. In the present *in vitro* study, (S)-(-)-blebbistatin O-benzoate inhibited the expression of *TSLP* and *IL-33* in keratinocytes under inflammatory conditions stimulated with antigens derived from mite excreta. Furthermore, *in vivo* application of (S)-(-)-blebbistatin O-benzoate significantly suppressed dermatitis symptoms, reduced serum IgE levels, and decreased the number of mast cells infiltrating the skin tissue. These results suggest that the type 2 immune response was suppressed by controlling the production of type 2 alarmin cytokines from keratinocytes in the lesions and that a series of inflammatory responses in AD were suppressed. Currently, biologics targeting TSLP and IL-33 are being developed and are considered promising therapeutic agents for AD [46, 47]. In the future, it is necessary to investigate how intracellular signaling is altered in order to elucidate the detailed mechanism of action of (S)-(-)-blebbistatin O-benzoate.

Based on the above findings, we speculate that (-)-blebbistatin and its analogs may suppress dermatitis symptoms by acting on multiple aspects of the pathogenesis of AD, including the formation of an epidermal barrier, suppression of type 2 immune cell induction, and suppression of immune cell migration. Therefore, these compounds may be developed as treatment options for AD to control dermatitis symptoms by suppressing inflammation and improving barrier function. Topical steroids, the most standard treatment option for AD, have excellent anti-inflammatory properties but adversely affect normal skin barrier homeostasis [48, 49]. In addition, it has been suggested that the control of inflammation with molecularly targeted agents such as dupilumab can normalize skin barrier formation and improve its function [50], but they do not act directly on keratinocytes to control factors involved in barrier formation [51]. Therefore, combining conventional treatment of AD with (S)-(-)-blebbistatin O-benzoate that actively upregulates skin barrier function could be a promising complementary treatment. NC/Nga mice are widely used as excellent pathological models that mimic the symptoms of human AD. However, it reflects only limited aspects of human AD symptoms and does not represent the complete pathology. Therefore, human clinical studies are essential to better understand the efficacy of (S)-(-)-blebbistatin O-benzoate in the treatment of AD.
